# Insights into the structure and architecture of the CCR4–NOT complex

**DOI:** 10.3389/fgene.2014.00137

**Published:** 2014-05-16

**Authors:** Kun Xu, Yuwei Bai, Aili Zhang, Qionglin Zhang, Mark G. Bartlam

**Affiliations:** ^1^State Key Laboratory of Medicinal Chemical Biology, Nankai UniversityTianjin, China; ^2^College of Life Sciences, Nankai UniversityTianjin, China

**Keywords:** CBR4–NOT complex, mRNA decay, poly(A), deadenylation, NOT module, protein structure

## Abstract

The CCR4–NOT complex is a highly conserved, multifunctional machinery with a general role in controlling mRNA metabolism. It has been implicated in a number of different aspects of mRNA and protein expression, including mRNA degradation, transcription initiation and elongation, ubiquitination, and protein modification. The core CCR4–NOT complex is evolutionarily conserved and consists of at least three NOT proteins and two catalytic subunits. The L-shaped complex is characterized by two functional modules bound to the CNOT1/Not1 scaffold protein: the deadenylase or nuclease module containing two enzymes required for deadenylation, and the NOT module. In this review, we will summarize the currently available information regarding the three-dimensional structure and assembly of the CCR4–NOT complex, in order to provide insight into its roles in mRNA degradation and other biological processes.

## INTRODUCTION

The CCR4–NOT is a highly conserved, multifunctional machinery with a general role in controlling mRNA metabolism (Collart and Panasenko, 2012). It catalyzes the deadenylation process, whereby the removal of mRNA poly(A) tails represses translation and marks the mRNA for degradation (Collart, 2003). Deadenylation, which is crucial for gene expression and is involved in biological processes ranging from embryonic development to learning and memory, is believed to be a biphasic process (Yamashita et al., 2005; Wahle and Winkler, 2013). The first phase involves synchronous and stepwise shortening of the poly(A) tail to ~110 nt. In the second phase, which is crucial for triggering decay of the mRNA body, the mRNAs become more heterogeneous in the lengths of their poly(A) tails, ranging from ~20 nt to ~110 nt. Several observations support the hypothesis that biphasic deadenylation is the result of the sequential action of PAN2–PAN3 and CCR4–NOT complexes, with PAN2–PAN3 dominating the first phase and CCR4–NOT the second phase (Yamashita et al., 2005; Zheng et al., 2008; Wahle and Winkler, 2013). The CCR4–NOT complex is involved in a number of other important biological processes, including transcription initiation and elongation, ubiquitination and protein modification (reviewed in Collart and Panasenko, 2012). Members of the CCR4–NOT complex are also associated with various functions in several species, either in the nucleus or the cytoplasm, including DNA repair and histone methylation in yeast, spindle positioning, and regulation of microtubule length in *Caenorhabditis elegans* and spermatogenesis in mice.

The core CCR4–NOT complex is highly and evolutionarily conserved among eukaryotes (Draper et al., 1995; Albert et al., 2000; Gavin et al., 2002). Early studies on the *Saccharomyces cerevisiae* complex by mass spectrometry and co-immunoprecipitation led to the identification of the CCR4–NOT complex approximately 0.9–1.2 MDa in size (Liu et al., 1998). The yeast core complex was found to contain five NOT proteins (Not1–Not5) and two catalytic subunits, Ccr4 and Caf1 (Pop2). In mammals, including humans, their homologs also form a similar multi-subunit complex which plays a significant role in the regulation of several cellular machines (Mahadevan and Struhl, 1990; Collart and Struhl, 1993, 1994; Lee et al., 1998; Oberholzer and Collart, 1998; Badarinarayana et al., 2000; Albert et al., 2002; Deluen et al., 2002; Lenssen et al., 2002; Maillet and Collart, 2002; Viswanathan et al., 2003). Additional subunits in the human CCR4–NOT complex include RQCD1 (also known as CNOT9/Not9/Rcd1/Caf40), Caf130, Not10 (CNOT10), C2orf29 (CNOT11), and TAB182, although some are species specific. For instance, Caf130 is specific to yeast (Collart and Panasenko, 2012), while Not10, C2orf29, and TAB182 in the human complex are not found in yeast (Lau et al., 2009) and Not10 and C2orf29 are conserved in metazoans (Bawankar et al., 2013; Mauxion et al., 2013).

Interaction studies have shown the *S. cerevisiae* complex is centered on the scaffold protein Not1, which is essential for yeast viability (Maillet et al., 2000). Similarly, human CNOT1 has been shown to be essential for viability of cells (Ito et al., 2011b), and is required for cell proliferation but not cell viability in MCF7 cells (Mittal et al., 2011). Not4 was shown to possess a functional RING finger domain in its N-terminal by NMR (Hanzawa et al., 2001), and its human homolog was subsequently confirmed to function as an E3 ligase by *in vitro* ubiquitination (Albert et al., 2002). Not2 contains no known functional motifs but has two functional domains: a C-terminal region involved in CCR4–NOT function, and an N-terminal domain that interacts with the protein Ada2 (Benson et al., 1998). Not3 and Not5 share similar N-terminal regions, but their respective functions remain unclear. The complex also contains two deadenylases (to be discussed in more detail below), as well as Caf40 and Caf130 with indeterminate function. A number of additional binding partners have been identified for the *S. cerevisiae* complex but will not be discussed further in this review (see Collart, 2003 for more details).

In contrast to the yeast CCR4–NOT complex, which is well-understood as a result of extensive yeast genetics studies, the human complex has been less well studied until relatively recently. Analysis of the human CCR4–NOT complex from stable HeLa cell derivatives expressing epitope-tagged CCR4–NOT subunits confirmed it to be generally similar to the yeast complex in composition, with CNOT1 as a scaffold and interacting with several CNOT proteins (CNOT2, CNOT7, CNOT8, and CNOT9; Lau et al., 2009). However, several differences were observed between the yeast and human complexes. For instance, multiple deadenylases are observed in human cells with different binding properties, although both human and yeast CCR4–NOT complexes contain only one Ccr4 and one Caf1 subunit. Whilst CNOT3 was not observed to bind directly to CNOT1 in yeast two-hybrid experiments, but instead binds to CNOT2 and integrates into the complex via this interaction, this view has been superseded by recent structural evidence (discussed below). Finally, CNOT4 is a true ortholog of yeast Not4p but, unlike its yeast counterpart, it appears not to be stably integrated into the human complex although it can interact with the scaffold protein CNOT1. More recently, the *Drosophila melanogaster* CCR4–NOT complex was shown to share a similar composition to the human CCR4–NOT complex (Temme et al., 2010; Bawankar et al., 2013). *D. melanogaster* NOT4, a homolog of human CNOT4, is also not stably integrated into the complex. Two additional components, NOT10 and NOT11/C2orf29, were identified to dock onto the NOT1 N-terminal domain and are conserved in both the human and *D. melanogaster* complexes (Bawankar et al., 2013; Mauxion et al., 2013).

Increasing numbers of studies on the properties of the CCR4–NOT complex has led to greater understanding about its three-dimensional structure and its precise roles in the cell. In recent years, the availability of information on the structure and architecture of the CCR4–NOT complex has increased dramatically. In this review, we focus on the structure and architecture of the multi-subunit and multifunctional CCR4–NOT complex, particularly the components involved in deadenylation and mRNA degradation and the more recently characterized NOT module. We aim to summarize the current state of structure-function studies pertaining to the CCR4–NOT components.

## ARCHITECTURE OF THE CCR4–NOT COMPLEX

**FIGURE 1:**
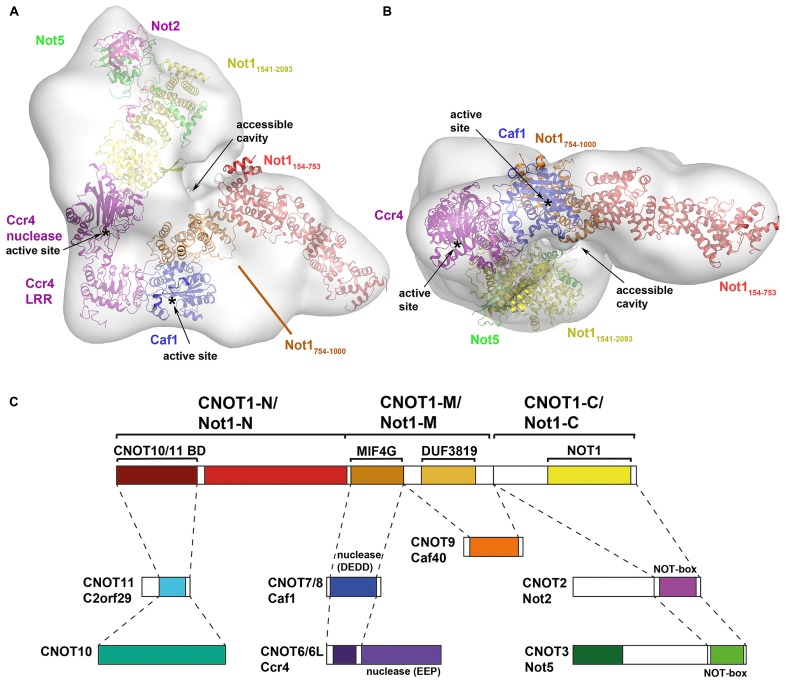
**Architecture of the CCR4–NOT complex. (A,B)** Model of the CCR4–NOT complex using X-ray crystal structures docked into a negative-stain EM map of the yeast CCR4–NOT complex (EMDB ID: 1901; Nasertorabi et al., 2011). Crystal structures of CCR4–NOT subunits were docked into the EM map using the UCSF Chimera software based on size and shape considerations and guided by the prediction of Nasertorabi and colleagues. Structures used for docking are: N-terminal domain of Not1 (PDB ID: 4B89); Not1–Ccr4–Caf1 (PDB ID: 4B8C); CNOT6L nuclease domain (PDB ID: 3NGQ); Not1–Not2–Not3 (PDB ID: 4BY6). The active sites of Ccr4 and Caf1 and the accessible cavity in the CCR4–NOT complex are labeled. **(C)** Map of the interactions in the CCR4–NOT complex (adapted from Bawankar et al., 2013). Subunits or domains are colored according to structures in **(A,B)** where available.

To date, two studies have provided detailed information about the overall architecture of the CCR4–NOT complex. In the first, a low-resolution electron microscopy (EM) reconstruction of the 1.0 MDa yeast CCR4–NOT complex isolated using Not1 as bait reveals an L-shaped structure with two arms of similar length (180–190 Å; Nasertorabi et al., 2011). The CCR4–NOT complex, including the subunits Not1–Not5, Ccr4, Caf1, Caf40, and Caf130, is natively heterogeneous and the authors were only able to obtain homogeneous L-shaped particles by chemical cross-linking of its components. Interestingly, using Caf1 as bait only yielded a Not1–Ccr4–Caf1 complex. The authors reported that relative arrangement of the arms varied between different reconstructions, indicating the whole assembly is flexible and therefore limiting resolution of the resulting EM map. Although positions of the subunits were not experimentally validated, prediction by the authors based on size consideration suggests that Not1–Ccr4–Caf1 is located in the hinge region connecting the two arms (**Figures 1A,B**), where an accessible cavity could provide a platform for interaction with RNA or regulating factors. The NOT module, which has been shown to affect the stability and deadenylase activity of the complex, is predicted to lie in the bulkier arm of the complex adjacent to the deadenylase module (**Figures 1A,B**), thus rationalizing how crosstalk can occur between the two modules. The N-terminal region of Not1 (154–753) would then extend into the thinner arm of the complex where it could interact with other subunits, such as Caf40 and Caf130 in the yeast complex or CNOT10 and CNOT11 in the mammalian complex. This arrangement of subunits in the CCR4–NOT complex is in good agreement with a model constructed with currently available structures of the deadenylase and NOT modules (**Figure 1**). Tob/BTG proteins bind directly to Caf1, while other regulators could be recruited to either the extended N-terminal HEAT-repeat platform or the C-terminal NOT module.

Bawankar et al. (2013) mapped the interactions of the individual subunits in the *Drosophila melanogaster* CCR4–NOT complex by co-immunoprecipitation and pull-down assays. The results not only confirmed the interactions that have been reported in various species, particularly the yeast and human complexes, they extended the interactions and provided possibly the most complete view of the CCR4–NOT complex to date. The results confirmed that NOT1 (homologous to CNOT1/Not1) is a scaffold protein for assembly of the complex, including the conserved NOT and deadenylase modules. The authors delineated three regions of NOT1 (CNOT1/Not1). The deadenylase module binds to the middle region via a MIF4G domain in NOT1 (CNOT1/Not1), with POP2 (CNOT7/Caf1) serving as an adaptor between NOT1 (CNOT1/Not1) and CCR4 (CNOT6L/Ccr4). A neighboring DUF3819 domain of unknown function in the middle region serves as the binding site for CAF40 (CNOT9/Caf40). The NOT module interacts with the C-terminal region of CNOT1/Not1. In their interaction map, NOT2 (CNOT2/Not2) interacts with NOT1 (CNOT1/Not1), whereas NOT3 (CNOT3/Not5) interacts with NOT2 (CNOT2/Not2) via their respective NOT boxes and has no direct interaction with NOT1 (CNOT1/Not1). Subsequent structural analysis has revised this view (see below). Two studies on the human and *D. melanogaster* complexes also showed that the N-terminal residues of CNOT1/Not1 interact with two proteins, CNOT10 (NOT10 in *D. melanogaster*) and CNOT11/C2orf29 (NOT11), which have no obvious orthologs in yeast (Mauxion et al., 2013; Bawankar et al., 2013). The functional significance of this CNOT10/CNOT11 module is unclear but, as it is not observed in yeast, it has been proposed to be an optional module or a functional homolog of the yeast Caf130 protein.

## THE CCR4–NOT DEADENYLASES

The CCR4–NOT complex includes two distinct deadenylase enzymes belonging to different families (see Bartlam and Yamamoto, 2010 for a review). The first are the DEDD-type nucleases, so named after the conserved catalytic Asp and Glu residues in three exonuclease motifs, including POP2, CAF1Z, poly(A)-specific ribonuclease (PARN), and PAN2. The second are the EEP-type nucleases, or exonuclease–endonuclease–phosphatase, including CCR4, Nocturnin, ANGEL, and 2′ phosphodiesterase (2′PDE). The CCR4–NOT complex is characterized by the presence of one deadenylase enzyme from each family. The yeast CCR4–NOT complex encompasses two deadenylases, Ccr4 and Caf1 (Pop2), both of which are involved in mRNA degradation, although Caf1 is dispensable for the deadenylase activity of Ccr4. The human CCR4–NOT complex is known to have two orthologs for each of the yeast deadenylases, Ccr4 and Caf1 (Bogdan et al., 1998; Albert et al., 2000; Dupressoir et al., 2001). The two human orthologs of yeast Ccr4 are CNOT6 (CCR4a) and CNOT6L (CCR4), which evidently have different functions as CNOT6L is involved in cell proliferation in mouse 3T3 fibroblasts but CNOT6 is not (Morita et al., 2007). This phenomenon appears to be cell-type specific, as both CNOT6 and CNOT6L are required for cell viability in MCF7 cells (Mittal et al., 2011). The two orthologs of Caf1 are CNOT7 (CAF1) and CNOT8 (POP2/CALIF), which share high sequence similarity, have partially overlapping function and are both important for cell proliferation (Aslam et al., 2009). Lau et al. (2009) identified distinct human CCR4–NOT complexes with either CNOT7 or CNOT8, implying that they compete for the same binding site on CNOT1. In the same study, CNOT6 and CNOT6L are mutually exclusive in CCR4–NOT complexes (Lau et al., 2009). Structures are presently available for representatives of each family and will be discussed in more detail below.

### DEDD-TYPE DEADENYLASES

**FIGURE 2:**
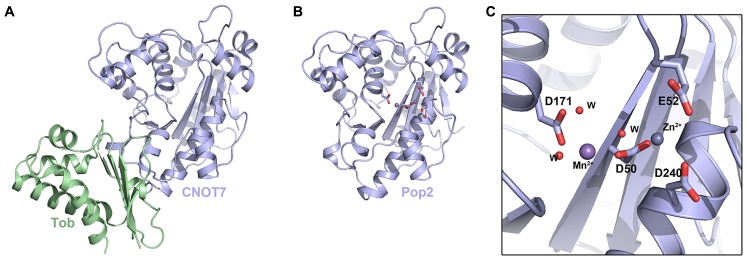
**The DEDD-type deadenylases. (A)** Crystal structure of the human CNOT7–Tob complex (PDB ID: 2D5R). **(B)** Crystal structure of *S. pombe* Caf1 (PDB ID: 3G0Z). **(C)** Enlarged view of the *S. pombe* Caf1 active site showing the DEDD motif in stick representation. Mn^2^^+^ and Zn^2^^+^ ions in the active site are shown as spheres and waters are shown as small red spheres.

The crystal structure of Caf1 was first determined from *S. cerevisiae* in 2003 (Thore et al., 2003), and then from *Schizosaccharomyces pombe* in 2007 (Jonstrup et al., 2007; Andersen et al., 2009; **Figure 2**). Caf1 adopts a kidney-shaped structure with 13 α-helices and 6 β-strands (**Figure 2A**). Comparison with related structures placed Caf1 as a member of the DEDD-type nucleases with the common fold of the DnaQ subgroup. DEDD-type nucleases are so named because they feature an Asp–Glu–Asp–Asp sequence motif in their active site. Despite low sequence similarity, Caf1 shares structural similarity with the ∊-subunit of DNA polymerase III and the exonuclease domain of DNA polymerase I.

The structure of *S. pombe* Caf1 provided insight into the selectivity and activity of this class of deadenylase (Jonstrup et al., 2007). Two divalent metal ions (sites A and B) located in the active site were found to be essential for activity (**Figures 2A,B**). Interestingly, although Mg^2^^+^ is found in higher concentrations inside cells, the A site of *S. pombe* Caf1 was found to favor Zn^2^^+^ and the B site exhibited a preference for Mn^2+^, despite a large 100-fold excess of Mg^2^^+^ in the cell. The composition of the two ions in the active site was also found to have an effect on the kinetics of deadenylation. In the presence of Mg^2^^+^, Mn^2^^+^, and Zn^2^^+^ ions, deadenylation was slow and unspecific, and only ~25% of RNA substrates were completely deadenylated after 160min. In the absence of Zn^2^^+^, however, Caf1 was observed to quickly and specifically degrade the complete poly(A) tail of the RNA substrate, suggesting that variations in cellular Zn^2^^+^ levels might provide a means to regulate the overall rate of mRNA turnover. The *S. cerevisiae* Caf1 revealed that the consensus DEDD nuclease motif is substituted by an SEDQ motif and so it lacks several essential highly conserved catalytic residues, implying it may have lost its catalytic activity and that its role might be architectural, such as to recruit Ccr4 into the CCR4–NOT complex. Caf1 was shown to function as an active exonuclease *in vitro* on monotonous RNA sequences, with a slight preference for poly(A) RNA over poly(U) and poly(C). This lack of absolute specificity suggests that Caf1 may not bind RNA substrates in a specific manner, but may involve a substantial contribution of van der Waals interactions.

The structure of human CNOT7, an ortholog of yeast Caf1, was reported in complex with the antiproliferative protein Tob (**Figure 2C**; Horiuchi et al., 2009). Unsurprisingly, CNOT7 shares high structural similarity with Caf1 from *S. pombe* and *S. cerevisiae*, as well as to a number of DEDD-type nucleases including human PARN. The CNOT7 structure was found to adopt the core catalytic domain of the RNase D superfamily, characterized by the DEDD sequence motif. These conserved acidic amino acids are responsible for metal ion binding. As with the *S. pombe* Caf1 structural study, Horiuchi et al. (2009) examined the nuclease activity of CNOT7 in the presence of the metal ions Mg^2^^+^, Ca^2^^+^, Mn^2^^+^, and Co^2^^+^. No activity was observed in the presence of Ca^2^^+^, and CNOT7 exhibited significantly higher activity for RNA substrates over DNA substrates. Highest RNase activity was observed in the presence of Mn^2^^+^, suggesting that Mn^2^^+^ is required for full activity of CNOT7 (Horiuchi et al., 2009). A recent structure of the human CNOT1–CNOT7 complex by Petit et al. (2012) showed two Mg^2^^+^ ions in the A and B sites of CNOT7, although the two metals are unusually close at 3.9 A due to additional coordination of the A site Mg^2^^+^ ion by Glu278, as opposed to 4.6 A between ions in *S. pombe* Caf1.

Tob, a regulator that promotes mRNA degradation, is not present in yeast but accumulating evidence indicates that the Tob/BTG family members are common binding partners of CNOT7 in the human CCR4–NOT complex (Mauxion et al., 2009; Winkler, 2010). The interaction of Tob with CNOT7 is largely hydrophobic and mediated by Box A and Box B regions in the N-terminal BTG domain that are conserved throughout the Btg/TOB family, as observed in structures of human BTG2 and mouse TIS21 (Yang et al., 2008). The C-terminal region of Tob features a conserved PAM2 motif required for interaction with the C-terminal domain of PABP, suggesting that Tob serves as an adaptor protein for the interaction between the CCR4–NOT complex and PABP (Ezzeddine et al., 2007; Funakoshi et al., 2007). This is in contrast to the PAN2–PAN3 complex, which interacts directly with PABP via a PAM2 motif in PAN3 (Siddiqui et al., 2007). Funakoshi et al. (2007) proposed a hypothesis in which recruitment of a PABP–mRNA complex to the CCR4–NOT complex via the Tob–CNOT7 interaction would cause translation to be interrupted by rapid degradation of the poly(A) tail, presumably by CNOT6 or CNOT6L, followed by repression of cell growth.

### EEP-TYPE DEADENYLASES

**FIGURE 3:**
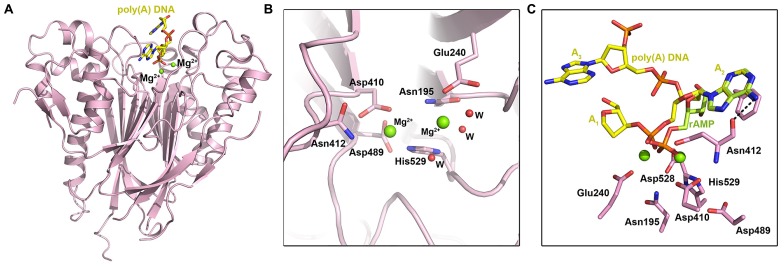
**The EEP-type deadenylases. (A)** Crystal structure of the human CCR4–NOT nuclease domain in complex with poly(A) DNA (PDB ID: 3NGO). The DNA ligand is shown in yellow stick representation and Mg^2^^+^ ions are shown as spheres. **(B)** Enlarged view of the human CNOT6L active site showing active site residues in stick representation. Mg^2^^+^ ions are shown as green spheres and waters are shown as small red spheres. **(C)** Substrate binding by human CNOT6L, showing poly(A) DNA (yellow stick representation) and rAMP (pale green stick representation) in the substrate binding pocket. CNOT6L active site residues are shown in pink and Mg^2^^+^ ions are shown as green spheres.

Ccr4 was identified to be the main deadenylase in yeast by the Denis (Malvar et al., 1992; Draper et al., 1994; Chen et al., 2002) and Parker (Tucker et al., 2001) labs. To date, the only structure of an intact Ccr4 nuclease domain is that of human CNOT6L (Wang et al., 2010), although a partial structure of the yeast Ccr4 nuclease domain has been reported as part of the Not1–Ccr4–Caf1 ternary complex (Basquin et al., 2012). The heart-shaped fold of CNOT6L features a two-layered α–β sandwich: two interior β-sheets are sandwiched between outer layers of α-helices (**Figure 3A**). A prominent active-site pocket at the top of the molecule features two bound Mg^2^^+^ ions (**Figures 3A,B**). Within the active site, the indispensable residue Glu240 coordinates the first, stable ion; mutation of Glu240 or absence of Mg^2^^+^ abolished enzymatic activity, showing that Mg^2^^+^ is a necessary cofactor (**Figure 3B**). Asp410, Asn412, and His529 coordinate the second, weakly bound magnesium ion. Subsequent experiments confirmed that the deadenylase activity of CNOT6L is Mg^2^^+^-dependent (Wang et al., 2010).

Deadenylase activity assays performed on poly(A), poly(G), poly(C), and poly(U) RNA substrates indicated that CNOT6L has strict substrate specificity for poly(A) RNA substrates, while similar experiments comparing activity for poly(A) RNA and DNA substrates revealed only trace activity for poly(A) DNA (Wang et al., 2010). Structural studies of CNOT6L with ribo-adenine-5′-monophosphate (rAMP) and a 5A stretch of poly(A) DNA were employed to probe the structural basis for this strict substrate specificity. In the poly(A) DNA structure, a 3A trinucleotide substrate was observed in the binding site with the scissile phosphate of A_2_ fixed by three bonds: two coordinated bonds with the Mg^2^^+^ ions and a third between Asn412 and the dissociative O atom of the phosphate moiety (**Figure 3C**). The adenine base of A_2_ stacks between the phenyl ring of Phe484 and the ring of Pro365, while a further bond is formed between the 6′-NH2 group of the adenine base and Asn412. A near-identical conformation of rAMP indicates the importance of Pro365, Asn412, and Phe484 in substrate recognition (**Figure 3C**): mutating the latter two abolished activity, while mutating Pro365 reduced activity. The purine base G, which shares the greatest similarity with A, has a carbonyl oxygen in the 6′ position that would clash with Asn412, while the pyrimidine base and 4′ –NH_2_ or carbonyl group of C or U bases could not be accommodated within the recognition pocket. Nucleotides A_1_ and A_3_ in the poly(A) DNA complex do not form specific interactions with CNOT6L.

## THE DEADENYLASE MODULE

**FIGURE 4:**
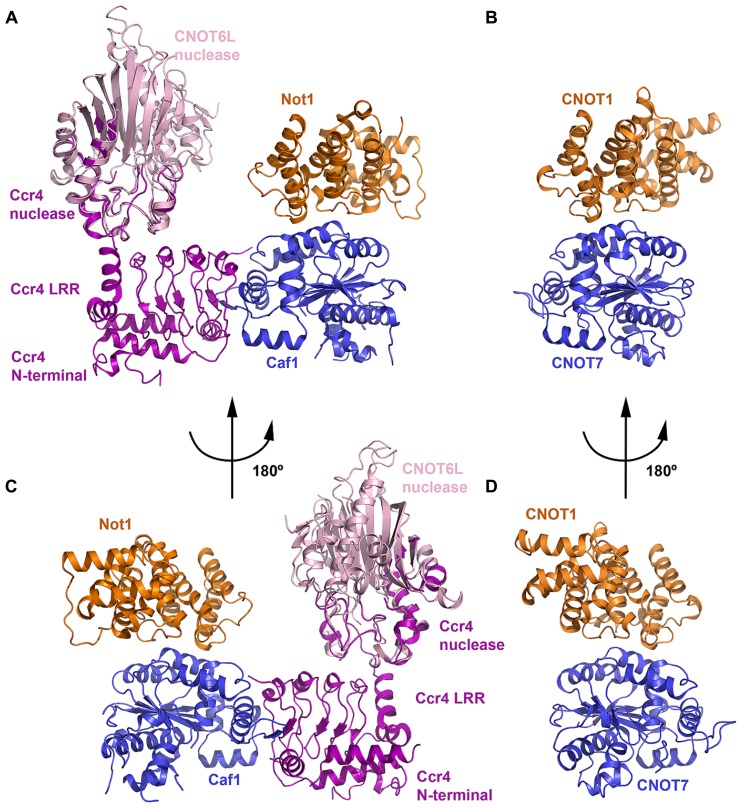
**The deadenylase module. (A)** Crystal structure of the yeast Not1–Ccr4–Caf1 ternary complex (PDB ID: 4B8C). The Not1 MIF4G domain is shown in orange, Caf1 in blue and Ccr4 in purple. The crystal structure of the human CNOT6L nuclease domain (light pink; PDB ID: 3NGQ) is shown superimposed onto the partial nuclease domain of Ccr4. **(B)** Crystal structure of the human CNOT1–CNOT7 binary complex (PDB ID: 4GMJ). The CNOT MIF4G domain (orange) and CNOT7 (blue) are colored according to their orthologs in **(A)**. The view shown is the same orientation as **(A)**. **(C)** View of the Not1–Ccr4–Caf1 ternary complex related to the view in panel A by a rotation of 180°. **(D)** View of the CNOT1–CNOT7 binary complex related to the view in **(B)** by a rotation of 180°.

The first structural view of a complete CCR4–NOT nuclease or deadenylase module was reported in 2012 for the yeast complex (Basquin et al., 2012; **Figure 4**). The ternary complex structure is formed between the N-terminal arm of Not1, Ccr4, and Caf1. The N-terminal arm of Not1 (residues 754–1000) forms a structure of five HEAT repeats related to the MIF4G (middle domain of initiation factor 4G) fold. Each HEAT motif consists of two antiparallel helices, termed A and B helices, and successive HEAT motifs pack together in an almost parallel manner. The CNOT1 MIF4G domain features a concave surface with a large patch of conserved residues contributed by the B helices of HEAT motifs 1-5. Another small patch of conserved residues is present on the opposite surface located in the interrepeat loops between HEAT3-4 and HEAT4-5. Although no structure exists for the complete vertebrate CCR4–NOT deadenylase module, Petit et al. (2012) reported a structure of the binary complex between human CNOT1–MIF4G (residues 1093–1317) and CNOT7.

In both human and yeast structures, CNOT7/Caf1 is recognized by and interacts with the small conserved hydrophobic patch in the CNOT1/Not1 MIF4G domain formed by the HEAT3-4 and HEAT4-5 inter-repeat loops (**Figure 4**). CNOT7/Caf1 binds CNOT1/Not1 at the opposite side to the active-site pocket, such that the active site is solvent exposed in the ternary complex. Only 8.4% of total accessible solvent area is buried in the Not1–Caf1 interface, leaving a large conserved patch of the Not1 concave surface accessible and exposed to solvent. Other regulatory factors may be recruited to the CCR4–NOT complex via this region. For instance, tristetraprolin (TTP) has been shown to bind to this MIF4G domain of CNOT1 (see below). Residues involved in the interaction between CNOT7/Caf1 and the CNOT1/Not1 MIF4G domain are primarily hydrophobic and are evolutionarily conserved.

Ccr4 interacts with Caf1 via its LRR (leucine rich repeat) domain (**Figures 4A,C**), which contains five repeats assembled into a crescent-shaped structure, but makes no direct interaction with Not1 (Basquin et al., 2012). The N-terminal region of Ccr4 interacts intermolecularly with the LRR domain, forming a single structural unit, while the 70 amino acid linker between the N-terminal region and LRR domain is disordered. In vertebrates, Ccr4 orthologs lack the N-terminal region and the concave surface of the LRR domain is polar. Half of the nuclease domain in the crystal structure is disordered, and the helices in the resolved half interact with the LRR domain. The interface between LRR and nuclease domains buries a surface area of about 400 Å^2^ and involves both hydrophobic and polar contacts. Superimposing the structure of the intact human CNOT6L nuclease domain (Wang et al., 2010) onto the partial Ccr4 nuclease domain shows that the active site does not contact Not1 and remains accessible.

Based on structural analysis, Basquin et al. (2012) studied the effects of mutants designed to disrupt the Not1–Caf1 and Caf1–Ccr4 interactions *in vivo*. While Ccr4–Caf1 are active deadenylases in the absence of Not1 *in vitro*, the *in vivo* results confirmed that incorporation of Caf1 and Ccr4 into the CCR4–NOT complex is essential for efficient deadenylation *in vivo*. Disrupting the association of Caf1 with Not1 resulted in a poor growth phenotype at high temperature, and mRNA decay intermediates accumulated when the ternary complex was disrupted. This is in good agreement with the observation by Petit et al. (2012) that a *D. melanogaster* CAF1 mutant that does not interact with NOT1 has a dominant-negative effect on mRNA degradation, inhibiting it by possibly sequestering CCR4 and preventing its incorporation into the endogenous CCR4–NOT complex. An intact complex may therefore be required for full efficient deadenylase activity, as the complex might target specific mRNA substrates or additional CCR4–NOT subunits may contribute to the activity. For instance, depleting CNOT2 is known to disrupt stability of the CCR4–NOT complex and inhibit its deadenylase activity, leading to apoptosis (Ito et al., 2011a).

## THE NOT MODULE

The second major module in the CCR4–NOT complex is formed by CNOT1, CNOT2, and CNOT3 in the human complex, and by Not1, Not2, and either Not3 or Not5 in the yeast complex. Both CNOT2 and CNOT3 in the human complex possess a NOT-box motif that has been shown to mediate their interaction (Zwartjes et al., 2004; Bawankar et al., 2013). In the yeast complex, Not2 is an ortholog of CNOT2, while Not3 and Not5 are orthologs of CNOT3 and thought to be paralogous proteins resulting from a gene duplication event (Collart et al., 2013). Sharing similar N-termini but distinct C-termini, Not5 is critically important for yeast vegetative growth whereas Not3 has only very mild phenotypes (Oberholzer and Collart, 1998). Whilst Not3 is predicted to have a similar fold and dimerization interface as Not2 and Not5, it does not bind to a Not2-ΔN mutant in contrast to Not5 and a subset of the dimerization interface diverges between the two paralogs (Bhaskar et al., 2013). The precise function of the NOT module remains unclear, but it has been linked to regulation of the CCR4–NOT complex stability and activity, and to recruitment of specific mRNAs (Wahle and Winkler, 2013). It has also been linked to poly(A) tail shortening, but its overall involvement in mRNA degradation is unclear. The NOT module also has distinct functions from the deadenylase module (Bai et al., 1999): for instance, it has been linked to transcription machinery [see (Collart and Panasenko, 2012; Miller and Reese, 2012) for reviews] and cytoplasmic mRNA decay pathways (Muhlrad and Parker, 2005; Temme et al., 2010).

**FIGURE 5:**
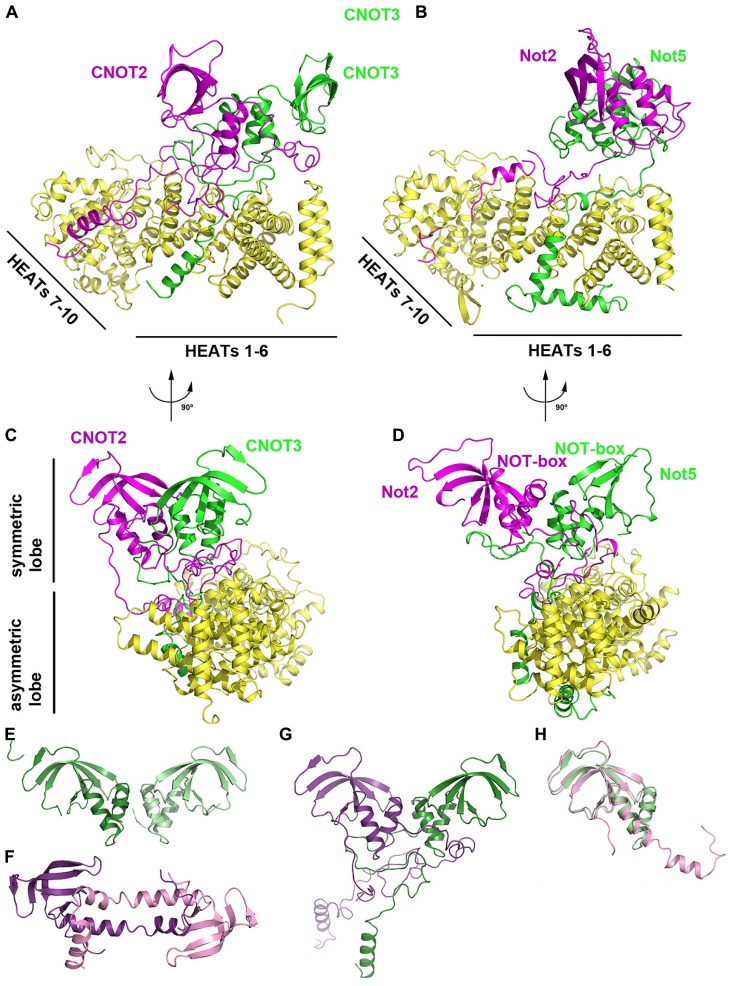
**The NOT module. (A)** Crystal structure of the human CNOT1–CNOT2–CNOT3 ternary complex (PDB ID: 4C0D). CNOT1 is shown in yellow, CNOT2 in magenta and CNOT3 in green. **(B)** Crystal structure of the yeast Not1–Not2–Not5 ternary complex (PDB ID: 4BY6). Not1 is shown in yellow, Not2 in magenta and Not5 in green. The complex is shown in the same orientation as **(A)**. **(C)** View of the human CNOT1–CNOT2–CNOT3 complex related by 90° to the orientation in **(A)**. **(D)** View of the human Not1-Not2-Not3 complex related by 90° to the orientation in **(B)**. **(E)** Crystal structure of the human CNOT3 NOT-box homodimer (PDB ID: 4C0G). **(F)** Crystal structure of the human CNOT2 NOT-box homodimer (PDB ID: 4C0F). **(G)** The CNOT2–CNOT3 heterodimer (PDB ID: 4C0D) shown in the same orientation as panel E. CNOT2 is shown in purple and CNOT3 is shown in green. **(H)** Superposition of the CNOT2 (PDB ID: 4C0F; pink) and CNOT3 (PDB ID: 4C0G; green) NOT-box structures.

Recent three-dimensional structures of the human CNOT1–CNOT2–CNOT3 (Boland et al., 2013) and yeast Not1–Not2–Not5 (Bhaskar et al., 2013) complexes have revealed the structure and assembly of the NOT module (**Figure 5**). Each structure shares a similar architecture in which CNOT2/Not2 and CNOT3/Not5 form a symmetric heterodimer via their NOT-box motifs, referred to as the symmetric lobe (**Figures 5A–D**). Extended N-terminal sequences, termed connector sequences (CSs) by Boland et al. (2013), extend the heterodimer interface by wrapping around the NOT-box of their neighbor. As the CSs of CNOT2/Not2 and CNOT3/Not5 differ in length and sequence, it is proposed that this structural divergence favors heterodimerization over homodimerization. Boland et al. (2013) observed that free CNOT2 and CNOT3 constructs containing both NOT-box and CSs tend to aggregate and precipitate in isolation, indicating that CSs are not favorable for homodimerization.

The heterodimer between CNOT2/Not2 and CNOT3/Not5 is tightly anchored to CNOT1/Not1 via unstructured, extended peptide regions, referred to as the asymmetric lobe (**Figure 5**). The C-terminal region of CNOT1/Not1 that forms the NOT module consists of 10 HEAT repeats, each characterized by a helix A-turn-helix B motif. The 10 HEAT repeats in the CNOT1/Not1 C-terminal region can be further divided into two units: HEAT motifs 1–6, and HEAT motifs 7–10 (**Figure 5**). The short CNOT3/Not5 anchor wraps around HEAT motifs 1–5 and interacts via hydrophobic and polar residues. The long CNOT2/Not2 anchor region zigzags across the surface of CNOT1/Not1, starting at HEAT motifs 9–10 and interacting with HEAT motifs 4–6. Notably, a conserved hydrophobic pocket in yeast Not1 formed by Leu2027, Leu2031, Phe2064, and Ile2071 recognizes Leu9 of Not2 (Bhaskar et al., 2013). An L9P mutant of Not2 results in loss of integrity of the *S. cerevisiae* CCR4–NOT complex, highlighting its functional importance *in vivo* (Russell et al., 2002).

The NOT-boxes of CNOT2/Not2 and CNOT3/Not5 are contained within their globular C-terminal domains. The NOT-box structure consists of three N-terminal α-helices and a C-terminal β-sheet formed by four (yeast) or five (human) β-strands, with considerable bending of strands β3/β4 (yeast) or β4/β5 (human). The NOT-boxes are most similar to Sm domains, but differ in that they lack signature Sm sequence motifs and lack an additional β-strand that mediates Sm–Sm dimerization. The NOT-box domains of CNOT2/Not2 and CNOT3/Not5 mediate dimerization via their N-terminal helices, resulting in a highly symmetrical heterodimer that adopts the same arrangement as a CNOT3 homodimer (**Figure 5E**; Boland et al., 2013) albeit with greater complementarity. A CNOT2 homodimer structure features domain swapping via the N-terminal helices that results in a distinct mode of dimerization from the CNOT3 homodimer and the CNOT2/CNOT3 heterodimer (**Figures 5E–G**). The NOT-box of CNOT3/Not5 is highly similar to that of CNOT2/Not2 and can be superimposed with an r.m.s.d. of <1.3 Å (**Figure 5H**).

Bhaskar et al. (2013) further revealed that the ternary Not1–Not2–Not5 complex forms a surface that binds poly(U) RNA *in vitro*, with mass spectrometry analysis identifying a nucleotide-binding site in Not5 centered around Cys546, different from the canonical nucleotide binding site of Sm folds. A U15 RNA substrate did not bind to Not1 or Not2–Not5 in isolation, suggesting that the three subunits cooperate to bind nucleotides, and the structure reveals a surface patch adjacent to Cys546 at the interface between Not1, Not2, and Not5. RNase protection assays used to estimate the length of the RNA-binding path on the complex indicated the accumulation of 11–15 nucleotide fragments, spanning a distance of 45–60 A. Mouse CNOT3 has previously been shown to regulate deadenylation of specific mRNAs via recruitment of their 3′ UTRs, which contain U-rich sequences (Morita et al., 2011). In yeast, Edc1 mRNA decay occurs via a deadenylation-independent pathway involving CCR4–NOT components and a poly-U tract in the 3′ UTR.

Based on their structural analysis, Boland et al. (2013) examined the integrity of the NOT module and its effects on mRNA degradation. In *D. melanogaster*, wild-type Not1 had no effect on mRNA half-life whereas a ΔN-SD mutant resulted in a two-fold increase in mRNA half-life. The ΔN-SD Not1 mutant was still able to bind Caf1, leading the authors to suggest that the catalytic module is sequestered into inactive or defective complexes that are no longer recruited to the mRNA reporter. Depletion of individual Not1, Not2, and Not3 subunits co-depleted the other two subunits, demonstrating strict control of respective protein ratios in the cell. Not1- and Not3-depleted cells also resulted in a ten-fold increase of hsp70 mRNA half-life relative to control cells. Not1 and Not3 mutants were also unable to restore mRNA degradation in Not1- and Not3-depleted cells in complementation assays. Taken together, they showed that an intact NOT module is essential for activity and recruitment of the CCR4–NOT complex and therefore for mRNA degradation.

## OTHER STRUCTURES

**FIGURE 6:**
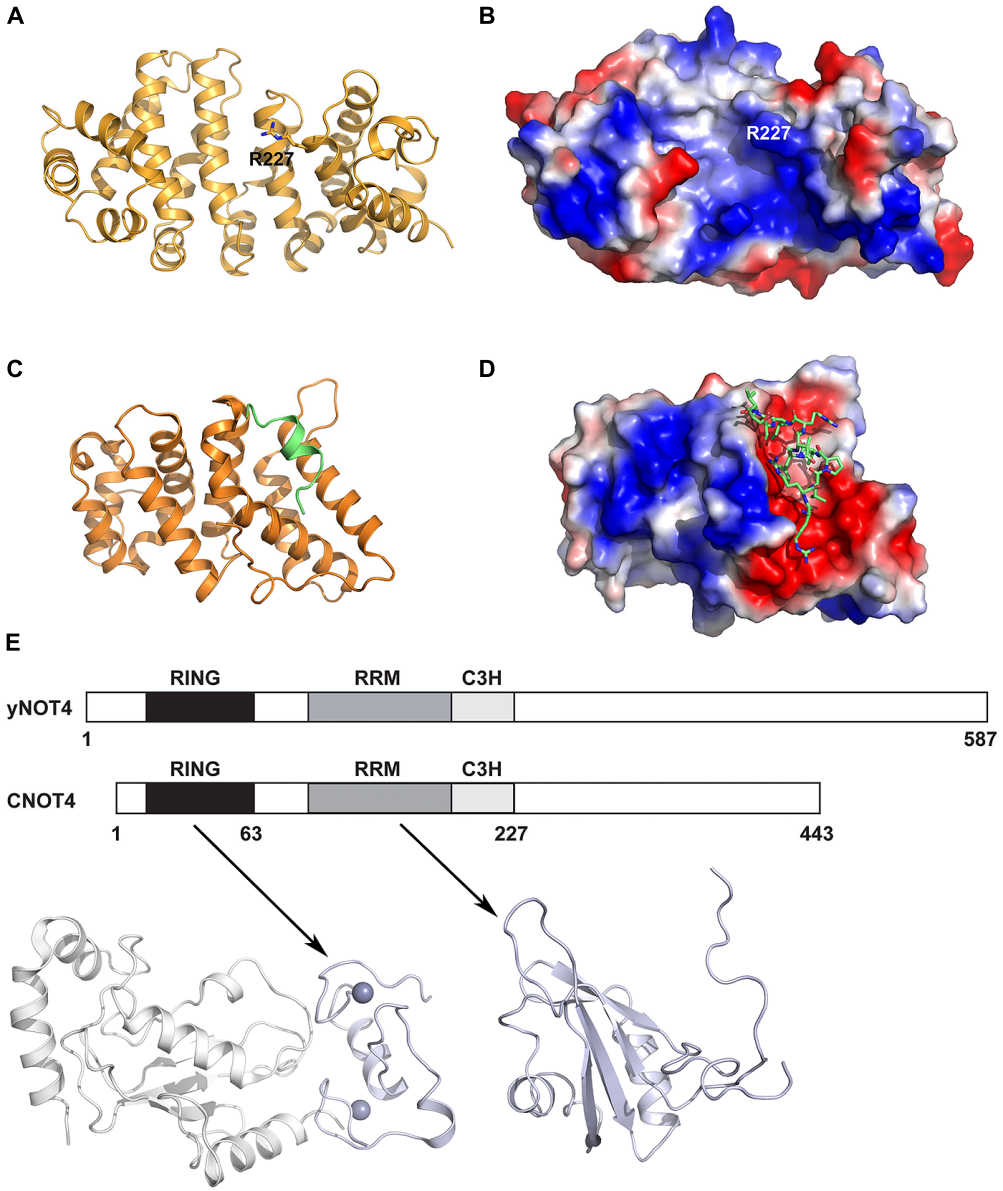
**Other CCR4–NOT protein structures. (A)** Crystal structure of human CNOT9 (PDB ID: 2FV2). The amino acid Arg227 implicated in nucleic acid binding is shown in stick representation. **(B)** Electrostatic surface representation of human CNOT9 showing the position of Arg227 in a region of positive charge. **(C)** Crystal structure of the human CNOT1 MIF4G domain in complex with a TTP peptide (PDB ID: 4J8S). The CNOT1 MIF4G domain is colored orange and the TTP peptide is colored green. **(D)** Electrostatic surface representation of the CNOT1 MIF4G domain showing the binding surface for the TTP peptide, shown in green stick representation. **(E)** The domain organization of yeast Not4 and human CNOT4. Shown below are the structural model of the human CNOT4 RING–UbcH5b complex (left; PDB ID: 1UR6) and the solution structure of the RRM (right, PDB ID: 2CPI) domain. The CNOT4 RING and RRM domains are shown in pale blue, and UbcH5b is shown in white.

In addition to the deadenylase and NOT modules, the CCR4–NOT complex includes several other subunits that are either peripheral to the core complex or species specific. The CNOT9/Rcd1 subunit (also known as RQCD1 or Caf40) has been shown to interact with CNOT1/Not1 via a central DUF3819 domain of unknown function (**Figure 1**; Bawankar et al., 2013). The crystal structure of CNOT9 has been determined and reveals an Armadillo repeat formed by a bundle of 18 α-helices arranged in a clockwork spiral (**Figures 6A,B**; Garces et al., 2007). The Armadillo motif is found in many diverse proteins with unrelated functions and, when folded together into a superhelix, provides a versatile platform for protein–protein interactions. CNOT9 was previously shown to interact with poly(G), poly(C), and poly(T) oligonucleotides, but was reported to have little or no detectable affinity for poly(A) sequences (Garces et al., 2007). The authors observed a positively charged cleft on the surface of CNOT9 and identified Arg227 in the cleft as important for nucleotide binding. The functional significance of this oligonucleotide binding remains unclear. However, as CNOT9 appears to be located in close proximity to the deadenylase module in the CCR4–NOT complex (Nasertorabi et al., 2011; Bawankar et al., 2013), it may contribute in some way to the deadenylase activity.

Tristetraprolin (TTP) is an RNA-binding protein that interacts with the CCR4–NOT complex to post-transcriptionally repress gene expression (Sandler et al., 2011). It achieves this by interacting with AU-rich elements (AREs) in 3′ untranslated regions of target mRNAs and subsequently engenders their deadenylation and decay. Fabian et al. (2013) identified an evolutionarily conserved C-terminal motif in human TTP that binds to the central region of CNOT1. A subsequent crystal structure reveals that a TTP peptide from residues 312–326 binds into a conserved hydrophobic groove between helices α1 and α3 of the CNOT1 MIF4G domain from residues 820–999 (**Figures 6C,D**). This is in good agreement with the finding by Sandler et al. (2011) that the CNOT1 region from amino acids 700–997 is important for the interaction with TTP. The central portion of the peptide forms a short two-turn amphipathic α-helix that binds into the groove, while the termini of the peptide forms a network of electrostatic interactions with surrounding negatively charged residues in CNOT1. An *in vitro* assay for TTP-mediated deadenylation showed that a mutant of TTP that could not bind to CNOT1 displayed severely impaired deadenylation, demonstrating that the TTP–CNOT1 interaction is required for TTP-mediated deadenylation *in vitro*. Similarly, TTP was found to require its C-terminal CNOT1-interacting motif for efficient decay of a target mRNA *in vivo*. The miRNA-associated protein GW182, which also binds to the CCR4–NOT complex via two CIMs, is believed to bind to different regions of CNOT1, as the TTP and GW182 CIMs share no homology (Fabian et al., 2011, 2013).

One key difference between yeast and vertebrate CCR4–NOT complexes is the CNOT4/Not4 protein, which has been shown to function as an E3 ubiquitin ligase. In yeast, Not4 is a stable member of the CCR4–NOT complex and interacts with Not1 via its C-terminus (Albert et al., 2002; Panasenko and Collart, 2011). In human cells, the homologous CNOT4 does not bind stably to CNOT1 and is only recruited to the CCR4–NOT complex under certain unspecified conditions (Lau et al., 2009), indicating that some functions of CNOT4 may be performed outside the complex. However, the Not4 function is conserved as human CNOT4 can partially compensate for the absence of the yeast protein (Albert et al., 2000). The yeast and human proteins share similar N-terminal regions (44% sequence identity over the first 230 amino acids of CNOT4) but divergent C-terminal regions, possibly reflecting their different associations with the CCR4–NOT complex (**Figure 6E**). The NMR structure of the CNOT4 RING domain has been determined and a structural model of the CNOT4 RING domain has been reported in complex with its E2 conjugating enzyme partner UbcH5B (Hanzawa et al., 2001; Dominguez et al., 2004), revealing the structural basis for E2/E3 specificity (**Figure 6E**). A solution structure has also been determined for the CNOT4 RNA-recognition motif (RRM, PDB ID: 2CPI; unpublished, **Figure 6E**). No structures are available for the C-terminal of CNOT4 or Not4, which has no identifiable domains. Known substrates of Not4 include the nascent polypeptide-associated Egd complex, a ribosomal chaperone, the ribosomal protein Rps7a, and the demethylase Jhd2. More recently, Not4 has been shown to play a role in protein quality control via its involvement in proteasome assembly (Panasenko and Collart, 2011), and this role is distinct from the CCR4–NOT deadenylase activity (Halter et al., 2014).

## PERSPECTIVES

Significant progress has been made in recent years towards understanding the three-dimensional structure and assembly of the CCR4–NOT complex. The interactions of the subunits in the core CCR4–NOT complex have been mapped and structures are now available for both the conserved deadenylase and NOT modules. Combining this wealth of structures at the atomic level with a low resolution negative staining EM map of the yeast CCR4–NOT complex facilitates modeling the CCR4–NOT complex (**Figure 1**), thus providing a more complete view of the complex. However, it should be stressed that such models are only a prediction and further structure-function studies are needed to elucidate the functional roles of the deadenylase and NOT modules and to study the mechanism of crosstalk between them.

Another important question to be addressed is why the deadenylase module contains two deadenylase enzymes, Ccr4 and Caf1. It is unclear how these enzymes cooperate and whether they play specialized or redundant roles (Winkler and Balacco, 2013). Both Ccr4 and Caf1 have a preference for poly(A) RNA substrates, but *S. cerevisiae* Caf1 has a SEDQ motif in place of the DEDD motif in its catalytic site and may not be active *in vivo*, serving only a structural role in recruiting Ccr4 to the complex (Basquin et al., 2012). In human cells, it is further unclear why two paralogs exist for Ccr4 (CNOT6, CNOT6L) and for Caf1 (CNOT7, CNOT8).

Whilst the structure and assembly of the core CCR4–NOT complex is better understood, little remains known about how various regulatory factors interact with the CCR4–NOT complex to regulate its multiple functions. It is also unclear how the CCR4–NOT complex targets specific mRNAs, which may involve a number of factors including the deadenylase module, the NOT module, poly(A)-binding protein PABP, translation factors and RNA-binding proteins [see (Collart and Panasenko, 2012) for a review]. Further structural studies will therefore be valuable in order to provide a more complete picture of the CCR4–NOT complex, to delineate the roles of its subunits and the interplay between them, and the association of the CCR4–NOT complex with various regulatory factors.

## Conflict of Interest Statement

The authors declare that the research was conducted in the absence of any commercial or financial relationships that could be construed as a potential conflict of interest.

## References

[B1] Albert T. K. Hanzawa H. Legtenberg Y. I. De Ruwe M. J. Van Den Heuvel F. A. Collart M. A. (2002). Identification of a ubiquitin-protein ligase subunit within the CBR4-NOT transcription repressor complex. *EMBO J.* 21 355–364 10.1093/emboj/21.3.355 11823428 PMC125831

[B2] Albert T. K. Lemaire M. Van Berkum N. L. Gentz R. Collart M. A. Timmers H. T. (2000). Isolation and characterization of human orthologs of yeast CBR4-NOT complex subunits. *Nucleic Acids Res.* 28 809–817 10.1093/nar/28.3.809 10637334 PMC102560

[B3] Andersen K. R. Jonstrup A. T. Van L. B. Brodersen D. E. (2009). The activity and selectivity of fission yeast Pop2p are affected by a high affinity for Zn2+ and Mn2+ in the active site. *RNA* 15 850–861 10.1261/rna.1489409 19307292 PMC2673079

[B4] Aslam A. Mittal S. Koch F. Andrau J. C. Winkler G. S. (2009). The CBR4-NOT deadenylase subunits CNOT7 and CNOT8 have overlapping roles and modulate cell proliferation. *Mol. Biol. Cell* 20 3840–3850 10.1091/mbc.E09-02-0146 19605561 PMC2735483

[B5] Badarinarayana V. Chiang Y. C. Denis C. L. (2000). Functional interaction of CBR4-NOT proteins with TATAA-binding protein (TBP) and its associated factors in yeast. *Genetics* 155 1045–1054 10880468 10.1093/genetics/155.3.1045 PMC1461164

[B6] Bai Y. Salvadore C. Chiang Y. C. Collart M. A. Liu H. Y. Denis C. L. (1999). The CBR4 and CAF1 proteins of the CBR4-NOT complex are physically and functionally separated from NOT2, NOT4, and NOT5. *Mol. Cell. Biol.* 19 6642–6651 10490603 10.1128/mcb.19.10.6642 PMC84645

[B7] Bartlam M. Yamamoto T. (2010). The structural basis for deadenylation by the CBR4-NOT complex. *Protein Cell* 1 443–452 10.1007/s13238-010-0060-8 21203959 PMC4875137

[B8] Basquin J. Roudko V. V. Rode M. Basquin C. Seraphin B. Conti E. (2012). Architecture of the nuclease module of the yeast CBR4-not complex: the Not1-Caf1-CBR4 interaction. *Mol. Cell* 48 207–218 10.1016/j.molcel.2012.08.014 22959269

[B9] Bawankar P. Loh B. Wohlbold L. Schmidt S. Izaurralde E. (2013). NOT10 and C2orf29/NOT11 form a conserved module of the CBR4-NOT complex that docks onto the NOT1 N-terminal domain. *RNA Biol.* 10 228–244 10.4161/rna.23018 23303381 PMC3594282

[B10] Benson J. D. Benson M. Howley P. M. Struhl K. (1998). Association of distinct yeast Not2 functional domains with components of Gcn5 histone acetylase and CBR4 transcriptional regulatory complexes. *EMBO J.* 17 6714–6722 10.1093/emboj/17.22.6714 9822614 PMC1171016

[B11] Bhaskar V. Roudko V. Basquin J. Sharma K. Urlaub H. Seraphin B. (2013). Structure and RNA-binding properties of the Not1-Not2-Not5 module of the yeast CBR4-Not complex. *Nat. Struct. Mol. Biol.* 20 1281–1288 10.1038/nsmb.2686 24121231

[B12] Bogdan J. A. Adams-Burton C. Pedicord D. L. Sukovich D. A. Benfield P. A. Corjay M. H. (1998). Human carbon catabolite repressor protein (CBR4)-associative factor 1: cloning, expression and characterization of its interaction with the B-cell translocation protein BTG1. *Biochem. J.* 336(Pt 2) 471–481 9820826 10.1042/bj3360471 PMC1219893

[B13] Boland A. Chen Y. Raisch T. Jonas S. Kuzuoglu-Ozturk D. Wohlbold L. (2013). Structure and assembly of the NOT module of the human CBR4-NOT complex. *Nat. Struct. Mol. Biol.* 20 1289–1297 10.1038/nsmb.2681 24121232

[B14] Chen J. Chiang Y. C. Denis C. L. (2002). CBR4, a 3^′^-5^′^ poly(A) RNA and ssDNA exonuclease, is the catalytic component of the cytoplasmic deadenylase. *EMBO J.* 21 1414–1426 10.1093/emboj/21.6.1414 11889047 PMC125924

[B15] Collart M. A. (2003). Global control of gene expression in yeast by the CBR4-Not complex. *Gene* 313 1–16 10.1016/S0378-1119(03)00672-3 12957374

[B16] Collart M. A. Panasenko O. O. (2012). The CBR4 – not complex. *Gene* 492 42–53 10.1016/j.gene.2011.09.033 22027279

[B17] Collart M. A. Panasenko O. O. Nikolaev S. I. (2013). The Not3/5 subunit of the CBR4-Not complex: a central regulator of gene expression that integrates signals between the cytoplasm and the nucleus in eukaryotic cells. *Cell. Signal.* 25 743–751 10.1016/j.cellsig.2012.12.018 23280189

[B18] Collart M. A. Struhl K. (1993). CDC39, an essential nuclear protein that negatively regulates transcription and differentially affects the constitutive and inducible HIS3 promoters. *EMBO J.* 12 177–186 8428577 10.1002/j.1460-2075.1993.tb05643.x PMC413189

[B19] Collart M. A. Struhl K. (1994). NOT1(CDC39), NOT2(CDC36), NOT3, and NOT4 encode a global-negative regulator of transcription that differentially affects TATA-element utilization. *Genes Dev.* 8 525–537 10.1101/gad.8.5.525 7926748

[B20] Deluen C. James N. Maillet L. Molinete M. Theiler G. Lemaire M. (2002). The CBR4-not complex and yTAF1 (yTaf(II)130p/yTaf(II)145p) show physical and functional interactions. *Mol. Cell. Biol.* 22 6735–6749 10.1128/MCB.22.19.6735-6749.2002 12215531 PMC134042

[B21] Dominguez C. Bonvin A. M. Winkler G. S. Van Schaik F. M. Timmers H. T. Boelens R. (2004). Structural model of the UbcH5B/CNOT4 complex revealed by combining NMR, mutagenesis, and docking approaches. *Structure* 12 633–644 10.1016/j.str.2004.03.004 15062086

[B22] Draper M. P. Liu H. Y. Nelsbach A. H. Mosley S. P. Denis C. L. (1994). CBR4 is a glucose-regulated transcription factor whose leucine-rich repeat binds several proteins important for placing CBR4 in its proper promoter context. *Mol. Cell. Biol.* 14 4522–4531 8007957 10.1128/mcb.14.7.4522-4531.1994 PMC358824

[B23] Draper M. P. Salvadore C. Denis C. L. (1995). Identification of a mouse protein whose homolog in *Saccharomyces cerevisiae* is a component of the CBR4 transcriptional regulatory complex. *Mol. Cell. Biol.* 15 3487–3495 7791755 10.1128/mcb.15.7.3487 PMC230585

[B24] Dupressoir A. Morel A. P. Barbot W. Loireau M. P. Corbo L. Heidmann T. (2001). Identification of four families of yCBR4- and Mg2+-dependent endonuclease-related proteins in higher eukaryotes, and characterization of orthologs of yCBR4 with a conserved leucine-rich repeat essential for hCAF1/hPOP2 binding. *BMC Genomics* 2:9. 10.1186/1471-2164-2-9 PMC61044 11747467

[B25] Ezzeddine N. Chang T. C. Zhu W. Yamashita A. Chen C. Y. Zhong Z. (2007). Human TOB, an antiproliferative transcription factor, is a poly(A)-binding protein-dependent positive regulator of cytoplasmic mRNA deadenylation. *Mol. Cell. Biol.* 27 7791–7801 10.1128/MCB.01254-07 17785442 PMC2169145

[B26] Fabian M. R. Cieplak M. K. Frank F. Morita M. Green J. Srikumar T. (2011). miRNA-mediated deadenylation is orchestrated by GW182 through two conserved motifs that interact with CBR4-NOT. *Nat. Struct. Mol. Biol.* 18 1211–1217 10.1038/nsmb.2149 21984185

[B27] Fabian M. R. Frank F. Rouya C. Siddiqui N. Lai W. S. Karetnikov A. (2013). Structural basis for the recruitment of the human CBR4-NOT deadenylase complex by tristetraprolin. *Nat. Struct. Mol. Biol.* 20 735–739 10.1038/nsmb.2572 23644599 PMC4811204

[B28] Funakoshi Y. Doi Y. Hosoda N. Uchida N. Osawa M. Shimada I. (2007). Mechanism of mRNA deadenylation: evidence for a molecular interplay between translation termination factor eRF3 and mRNA deadenylases. *Genes Dev.* 21 3135–3148 10.1101/gad.1597707 18056425 PMC2081979

[B29] Garces R. G. Gillon W. Pai E. F. (2007). Atomic model of human Rcd-1 reveals an armadillo-like-repeat protein with in vitro nucleic acid binding properties. *Protein Sci.* 16 176–188 10.1110/ps.062600507 17189474 PMC2203284

[B30] Gavin A. C. Bosche M. Krause R. Grandi P. Marzioch M. Bauer A. (2002). Functional organization of the yeast proteome by systematic analysis of protein complexes. *Nature* 415 141–147 10.1038/415141a 11805826

[B31] Halter D. Collart M. A. Panasenko O. O. (2014). The Not4 E3 Ligase and CBR4 Deadenylase play distinct roles in protein quality control. *PLoS ONE* 9:e86218. 10.1371/journal.pone.0086218 PMC3895043 24465968

[B32] Hanzawa H. De Ruwe M. J. Albert T. K. Van Der Vliet P. C. Timmers H. T. Boelens R. (2001). The structure of the C4C4 ring finger of human NOT4 reveals features distinct from those of C3HC4 RING fingers. *J. Biol. Chem.* 276 10185–10190 10.1074/jbc.M009298200 11087754

[B33] Horiuchi M. Takeuchi K. Noda N. Muroya N. Suzuki T. Nakamura T. (2009). Structural basis for the antiproliferative activity of the Tob-hCaf1 complex. *J. Biol. Chem.* 284 13244–13255 10.1074/jbc.M809250200 19276069 PMC2676056

[B34] Ito K. Inoue T. Yokoyama K. Morita M. Suzuki T. Yamamoto T. (2011a). CNOT2 depletion disrupts and inhibits the CBR4-NOT deadenylase complex and induces apoptotic cell death. *Genes Cells* 16 368–379 10.1111/j.1365-2443.2011.01492.x 21299754

[B35] Ito K. Takahashi A. Morita M. Suzuki T. Yamamoto T. (2011b). The role of the CNOT1 subunit of the CBR4-NOT complex in mRNA deadenylation and cell viability. *Protein Cell* 2 755–763 10.1007/s13238-011-1092-4 21976065 PMC4875264

[B36] Jonstrup A. T. Andersen K. R. Van L. B. Brodersen D. E. (2007). The 1.4-A crystal structure of the *S. pombe* Pop2p deadenylase subunit unveils the configuration of an active enzyme. *Nucleic Acids Res.* 35 3153–3164. 10.1093/nar/gkm178 17452359 PMC1888821

[B37] Lau N. C. Kolkman A. Van Schaik F. M. Mulder K. W. Pijnappel W. W. Heck A. J. (2009). Human CBR4-Not complexes contain variable deadenylase subunits. *Biochem. J.* 422 443–453 10.1042/BJ20090500 19558367

[B38] Lee T. I. Wyrick J. J. Koh S. S. Jennings E. G. Gadbois E. L. Young R. A. (1998). Interplay of positive and negative regulators in transcription initiation by RNA polymerase II holoenzyme. *Mol. Cell. Biol.* 18 4455–4462 9671455 10.1128/mcb.18.8.4455 PMC109031

[B39] Lenssen E. Oberholzer U. Labarre J. De Virgilio C. Collart M. A. (2002). *Saccharomyces cerevisiae* CBR4-not complex contributes to the control of Msn2p-dependent transcription by the Ras/cAMP pathway. *Mol. Microbiol.* 43 1023–1037 10.1046/j.1365-2958.2002.02799.x 11929548

[B40] Liu H. Y. Badarinarayana V. Audino D. C. Rappsilber J. Mann M. Denis C. L. (1998). The NOT proteins are part of the CBR4 transcriptional complex and affect gene expression both positively and negatively. *EMBO J.* 17 1096–1106 10.1093/emboj/17.4.1096 9463387 PMC1170458

[B41] Mahadevan S. Struhl K. (1990). Tc, an unusual promoter element required for constitutive transcription of the yeast HIS3 gene. *Mol. Cell. Biol.* 10 4447–4455 2201891 10.1128/mcb.10.9.4447-4455.1990 PMC361030

[B42] Maillet L. Collart M. A. (2002). Interaction between Not1p, a component of the CBR4-not complex, a global regulator of transcription, and Dhh1p, a putative RNA helicase. *J. Biol. Chem.* 277 2835–2842 10.1074/jbc.M107979200 11696541

[B43] Maillet L. Tu C. Hong Y. K. Shuster E. O. Collart M. A. (2000). The essential function of Not1 lies within the CBR4-Not complex. *J. Mol. Biol.* 303 131–143 10.1006/jmbi.2000.4131 11023781

[B44] Malvar T. Biron R. W. Kaback D. B. Denis C. L. (1992). The CBR4 protein from *Saccharomyces cerevisiae* contains a leucine-rich repeat region which is required for its control of ADH2 gene expression. *Genetics* 132 951–962 1459446 10.1093/genetics/132.4.951 PMC1205251

[B45] Mauxion F. Chen C. Y. Seraphin B. Shyu A. B. (2009). BTG/TOB factors impact deadenylases. *Trends Biochem. Sci.* 34 640–647 10.1016/j.tibs.2009.07.008 19828319 PMC2787745

[B46] Mauxion F. Preve B. Seraphin B. (2013). C2ORF29/CNOT11 and CNOT10 form a new module of the CBR4-NOT complex. *RNA Biol.* 10 267–276 10.4161/rna.23065 23232451 PMC3594285

[B47] Miller J. E. Reese J. C. (2012). CBR4-Not complex: the control freak of eukaryotic cells. *Crit. Rev. Biochem. Mol. Biol* 47 315–333 10.3109/10409238.2012.667214 22416820 PMC3376659

[B48] Mittal S. Aslam A. Doidge R. Medica R. Winkler G. S. (2011). The CBR4a (CNOT6) and CBR4b (CNOT6L) deadenylase subunits of the human CBR4-Not complex contribute to the prevention of cell death and senescence. *Mol. Biol. Cell* 22 748–758 10.1091/mbc.E10-11-0898 21233283 PMC3057700

[B49] Morita M. Oike Y. Nagashima T. Kadomatsu T. Tabata M. Suzuki T. (2011). Obesity resistance and increased hepatic expression of catabolism-related mRNAs in Cnot3+/- mice. *EMBO J.* 30 4678–4691 10.1038/emboj.2011.320 21897366 PMC3243589

[B50] Morita M. Suzuki T. Nakamura T. Yokoyama K. Miyasaka T. Yamamoto T. (2007). Depletion of mammalian CBR4b deadenylase triggers elevation of the p27Kip1 mRNA level and impairs cell growth. *Mol. Cell. Biol.* 27 4980–4990 10.1128/MCB.02304-06 17452450 PMC1951489

[B51] Muhlrad D. Parker R. (2005). The yeast EDC1 mRNA undergoes deadenylation-independent decapping stimulated by Not2p, Not4p, and Not5p. *EMBO J.* 24 1033–1045 10.1038/sj.emboj.7600560 15706350 PMC554118

[B52] Nasertorabi F. Batisse C. Diepholz M. Suck D. Bottcher B. (2011). Insights into the structure of the CBR4-NOT complex by electron microscopy. *FEBS Lett.* 585 2182–2186 10.1016/j.febslet.2011.05.071 21669201 PMC3171648

[B53] Oberholzer U. Collart M. A. (1998). Characterization of NOT5 that encodes a new component of the Not protein complex. *Gene* 207 61–69 10.1016/S0378-1119(97)00605-7 9511744

[B54] Panasenko O. O. Collart M. A. (2011). Not4 E3 ligase contributes to proteasome assembly and functional integrity in part through Ecm29. *Mol. Cell. Biol.* 31 1610–1623 10.1128/MCB.01210-10 21321079 PMC3126335

[B55] Petit A. P. Wohlbold L. Bawankar P. Huntzinger E. Schmidt S. Izaurralde E. (2012). The structural basis for the interaction between the CAF1 nuclease and the NOT1 scaffold of the human CBR4-NOT deadenylase complex. *Nucleic Acids Res.* 40 11058–11072 10.1093/nar/gks883 22977175 PMC3510486

[B56] Russell P. Benson J. D. Denis C. L. (2002). Characterization of mutations in NOT2 indicates that it plays an important role in maintaining the integrity of the CBR4-NOT complex. *J. Mol. Biol.* 322 27–39 10.1016/S0022-2836(02)00707-6 12215412

[B57] Sandler H. Kreth J. Timmers H. T. Stoecklin G. (2011). Not1 mediates recruitment of the deadenylase Caf1 to mRNAs targeted for degradation by tristetraprolin. *Nucleic Acids Res.* 39 4373–4386 10.1093/nar/gkr011 21278420 PMC3105394

[B58] Siddiqui N. Mangus D. A. Chang T. C. Palermino J. M. Shyu A. B. Gehring K. (2007). Poly(A) nuclease interacts with the C-terminal domain of polyadenylate-binding protein domain from poly(A)-binding protein. *J. Biol. Chem.* 282 25067–25075 10.1074/jbc.M701256200 17595167

[B59] Temme C. Zhang L. Kremmer E. Ihling C. Chartier A. Sinz A. (2010). Subunits of the Drosophila CBR4-NOT complex and their roles in mRNA deadenylation. *RNA* 16 1356–1370 10.1261/rna.2145110 20504953 PMC2885685

[B60] Thore S. Mauxion F. Seraphin B. Suck D. (2003). X-ray structure and activity of the yeast Pop2 protein: a nuclease subunit of the mRNA deadenylase complex. *EMBO Rep.* 4 1150–1155 10.1038/sj.embor.7400020 14618157 PMC1326415

[B61] Tucker M. Valencia-Sanchez M. A. Staples R. R. Chen J. Denis C. L. Parker R. (2001). The transcription factor associated CBR4 and Caf1 proteins are components of the major cytoplasmic mRNA deadenylase in *Saccharomyces cerevisiae*. *Cell* 104 377–386 10.1016/S0092-8674(01)00225-2 11239395

[B62] Viswanathan P. Chen J. Chiang Y. C. Denis C. L. (2003). Identification of multiple RNA features that influence CBR4 deadenylation activity. *J. Biol. Chem.* 278 14949–14955 10.1074/jbc.M211794200 12590136

[B63] Wahle E. Winkler G. S. (2013). RNA decay machines: deadenylation by the CBR4-not and Pan2-Pan3 complexes. *Biochim. Biophys. Acta* 1829 561–570 10.1016/j.bbagrm.2013.01.003 23337855

[B64] Wang H. Morita M. Yang X. Suzuki T. Yang W. Wang J. (2010). Crystal structure of the human CNOT6L nuclease domain reveals strict poly(A) substrate specificity. *EMBO J.* 29 2566–2576 10.1038/emboj.2010.152 20628353 PMC2928688

[B65] Winkler G. S. (2010). The mammalian anti-proliferative BTG/Tob protein family. *J. Cell. Physiol.* 222 66–72 10.1002/jcp.21919 19746446

[B66] Winkler G. S. Balacco D. L. (2013). Heterogeneity and complexity within the nuclease module of the CBR4-Not complex. *Front. Genet.* 4:296. 10.3389/fgene.2013.00296 PMC3870282 24391663

[B67] Yamashita A. Chang T. C. Yamashita Y. Zhu W. Zhong Z. Chen C. Y. (2005). Concerted action of poly(A) nucleases and decapping enzyme in mammalian mRNA turnover. *Nat. Struct. Mol. Biol.* 12 1054–1063 10.1038/nsmb1016 16284618

[B68] Yang X. Morita M. Wang H. Suzuki T. Yang W. Luo Y. (2008). Crystal structures of human BTG2 and mouse TIS21 involved in suppression of CAF1 deadenylase activity. *Nucleic Acids Res.* 36 6872–6881 10.1093/nar/gkn825 18974182 PMC2588512

[B69] Zheng D. Ezzeddine N. Chen C. Y. Zhu W. He X. Shyu A. B. (2008). Deadenylation is prerequisite for P-body formation and mRNA decay in mammalian cells. *J. Cell Biol.* 182 89–101 10.1083/jcb.200801196 18625844 PMC2447901

[B70] Zwartjes C. G. Jayne S. Van Den Berg D. L. Timmers H. T. (2004). Repression of promoter activity by CNOT2, a subunit of the transcription regulatory CBR4-not complex. *J. Biol. Chem.* 279 10848–10854 10.1074/jbc.M311747200 14707134

